# Energy absorbed from double quantum dot-metal nanoparticle hybrid system

**DOI:** 10.1038/s41598-022-25765-3

**Published:** 2022-12-13

**Authors:** Haneen Akram, Muwaffaq Abdullah, Amin H. Al-Khursan

**Affiliations:** Nasiriya Nanotechnology Research Laboratory (NNRL), College of Science, University of Thi-Qar, Nasiriya, Iraq

**Keywords:** Biotechnology, Engineering, Materials science, Nanoscience and technology

## Abstract

This work proposes the double quantum dot (DQD)-metal nanoparticle (MNP) hybrid system for a high energy absorption rate. The structure is modeled using density matrix equations that consider the interaction between excitons and surface plasmons. The wetting layer (WL)-DQD transitions are considered, and the orthogonalized plane wave (OPW) between these transitions is considered. The DQD energy states and momentum calculations with OPW are the figure of merit recognizing this DQD-MNP work. The results show that at the high pump and probe application, the total absorption rate $({Q}_{tot})$ of the DQD-MNP hybrid system is increased by reducing the distance between DQD-MNP. The high ${Q}_{tot}$ obtained may relate to two reasons: first, the WL washes out modes other than the condensated main mode. Second, the high flexibility of manipulating DQD states compared to QD states results in more optical properties for DQD. The ${Q}_{DQD}$ is increased at a small MNP radius on the contrary to the ${Q}_{MNP}$ which is increased at a wider MNP radius. Under high tunneling, a broader blue shift in the ${Q}_{tot}$ due to the destructive interference between fields is seen and the synchronization between ${Q}_{MNP}$ and ${Q}_{DQD}$ is destroyed. ${Q}_{tot}$ for the DQD-MNP is increased by six orders while ${Q}_{DQD}$ is by eight orders compared to the single QD-MNP hybrid system. The high absorption rate of the DQD-MNP hybrid system comes from the transition possibilities and flexibility of choosing the transitions in the DQD system, which strengthens the transitions and increases the linear and nonlinear optical properties. This will make the DQD-MNP hybrid systems preferable to QD-MNP systems.

## Introduction

Significant interest has been shown in the study of energy absorption in semiconductor quantum dot (QD)-metal nanoparticle (MNP) complexes in the last few years^1–10^. The initial work of Zhang, Govorov, and Bryant showed that applying an electromagnetic field could significantly modify the energy absorption spectrum. It can shift energy, exhibit broadening, and even the nonlinear Fano effect. The QD was treated as a two-level quantum system, the MNP as a classical electromagnetic particle, assuming that the excitons have a dipole–dipole interaction with surface plasmons, so the interaction was handled quasi-static^1^.

MNP has outstanding optical characteristics led to a revolution in physics, chemistry, biology, and material sciences^11,12^. Their capacity to amplify and focus optical fields to spots much smaller than the diffraction limit stems from localized surface plasmons (LSP), i.e., the collective wavelike motion of free electrons on the MNP surface^13^. With bio-assembly, self-organized epitaxial quantum dot (QD) growth has progressed in lockstep. The optical quality of self-assembled QDs is excellent with atomically sharp optical lines^14^. This capability for nanocrystal or biomaterial assembly allows the fabrication of sophisticated hybrid superstructures with a strong and discrete optical response from excitons in QDs and plasmons in MNPs^15,16^.

Plasmonics leads to a large number of applications that can merge electronics and photonics at the nanoscale, such as nanoscale laser cavities (spaser)^17^, ultra-sensitive spectroscopy^18^, and optical nanocircuits^19–21^. There are now several extensions to the original study. Artuso and Bryant studied the energy absorption spectrum of the QD-MNP system for strong applied fields^2,3^. They showed that exciton-induced transparency, bistability, discontinuous response, and suppression might occur in various interaction regions. Yan et al.^4^ investigated the role of multipole effects in the energy absorption spectrum when excitons and plasmons interact. Subsequent studies looked at the implications of the applied field quantum nature on the energy absorption spectrum^5,6^ and evaluated the system by treating surface plasmons as a quantum energy continuum^7^. The literature has also published extensions of QDs classified as a three-tier system^8,9^. Recently, the energy absorption spectrum in a hybrid QD-MNP has also been analyzed by a combination of the nonlinear density matrix with the boundary element method for the electromagnetic calculations to account for the local fields of such metal nanostructures, including retardation effects and the interaction to all multipolar orders^10^.

Chen et al. show a high increment in the nonlinear properties by combining surface plasmon resonance with the nonlinear system. A six-order increment was detected in the two-photon absorption compared to the ordinary structures^22^. The same group decided 230 times increment in the generation rate of organic solar cells by using a plasmon-enhanced method^23^.

He and Zhu^24^ and Hakami and Zubairy^25^ propose two QDs on the two sides of MNP. So, the two QDs are not coupled electronically. In this work, the DQD-MNP structure is introduced for a higher energy absorption rate depending on the high linear and nonlinear optical properties in the DQD structure due to the flexibility in manipulating the carrier transitions between the DQD structure compared to a single QD structure^26^. Here, the energy absorption rate from the DQD-MNP hybrid system is discussed using the density matrix equations. The WL-DQD transitions and OPW between them, the DQD energy states, and momentum calculations with OPW are the figure of merit recognizing this DQD-MNP work. The results show that at the high pump, the highest contribution comes from ${Q}_{MNP}$. A broader blue shift at higher tunneling is seen. At low pumping field, the ${Q}_{DQD}$ is higher by more than one order than the ${Q}_{MNP}$. The broader QD size exhibits a high ${Q}_{tot}$. Compared to their single QD-MNP counterpart, ${Q}_{tot}$ and ${Q}_{MNP}$ are increased by six orders while ${Q}_{SQD}$ is reduced by ten orders. This will make the DQD-MNP hybrid system preferable to QD-MNP.

## Theory

### DQD-MNP structure

**Figure 1:**
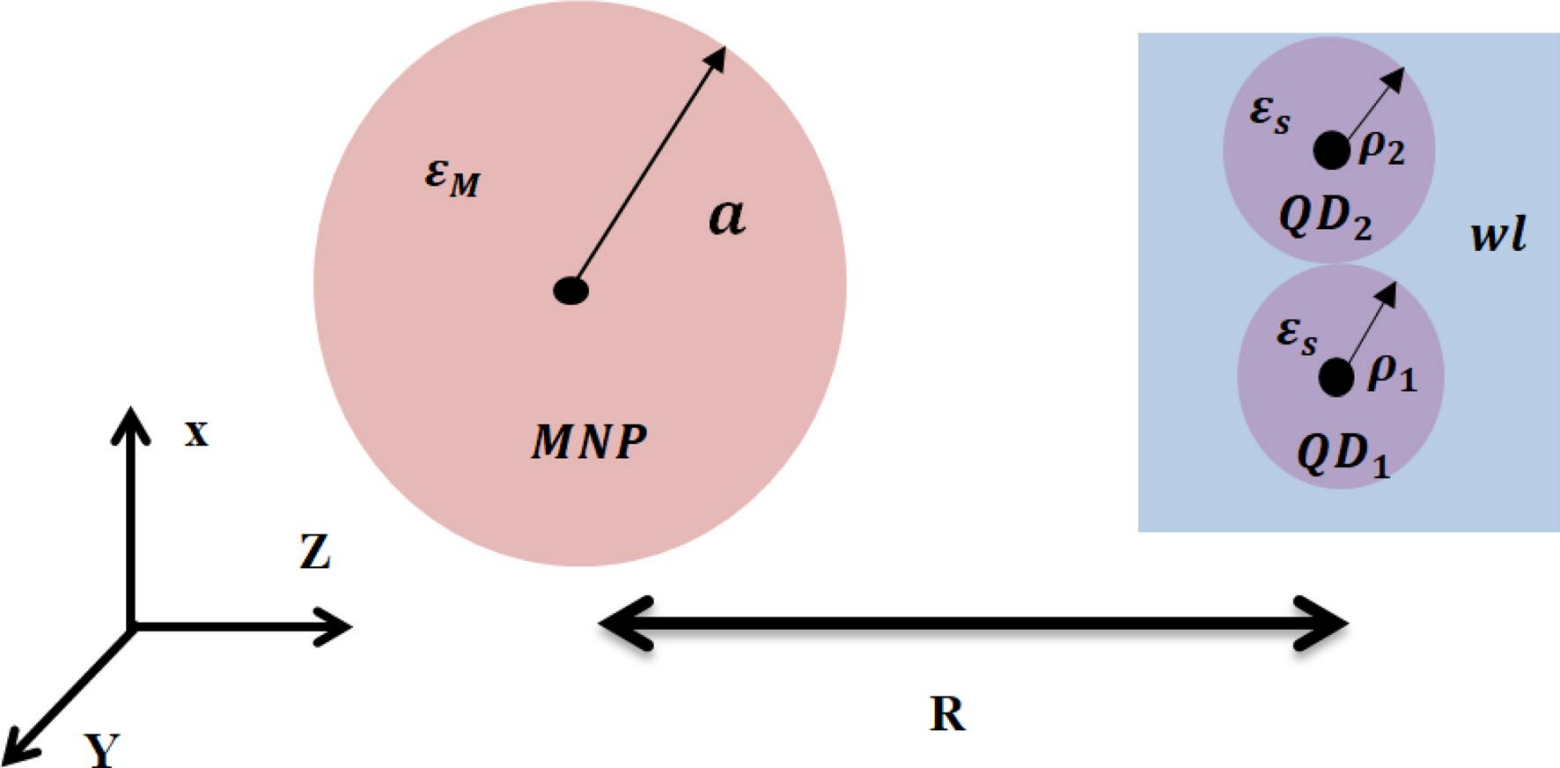
The hybrid DQD-MNP system. The separation between the centers of the two particles is R.

The hybrid structure studied here is composed of a DQD (the QDs are in a disk shape with radii $${\rho }_{1}, {\rho }_{2}$$) and a spherical MNP of radius ($$a$$) at interparticle distance ($$R)$$ (see Fig. 1) embedded in a material with a dielectric constant $${\varepsilon }_{B}$$. We also consider the radius of the DQD to be much smaller than that of the MNP, $$\left({\rho }_{1}, {\rho }_{2}<a\right)$$ and also $$\left(a<R\right)$$^27,28^. This system interacts with a linearly polarized oscillating electromagnetic field $$E\left(t\right)={E}_{0}cos\left(\omega t\right)$$ with $${E}_{0}$$ is the electric field amplitude, and ω is the angular frequency of the applied field. The DQD considered comprises two QDs; each QD was an InAs QD with a disk shape and height $${h}_{d}$$. The sizes of the first QD are ($${h}_{d1}$$ = 0.1 nm, $${\rho }_{1}$$ = 3 nm) while those of the second QD are ($${h}_{d2}=0.15 {\text{nm}}$$, $${\rho }_{2}=4 {\text{nm}}$$). Each QD has one conduction and valence subband. The wetting layer (WL) in the form of a quantum well is an InGaAs with 10 nm thickness, and their conduction and valence subbands work as reservoir states for both QDs. The structure is grown on a GaAs barrier. The dielectric constant of the QD is represented by $${\upvarepsilon }_{\mathrm{s}}$$ while the local dynamic dielectric function of the MNP is $${\upvarepsilon }_{\mathrm{M}}$$.

### The Hamiltonian of the DQD-MNP system

**Figure 2:**
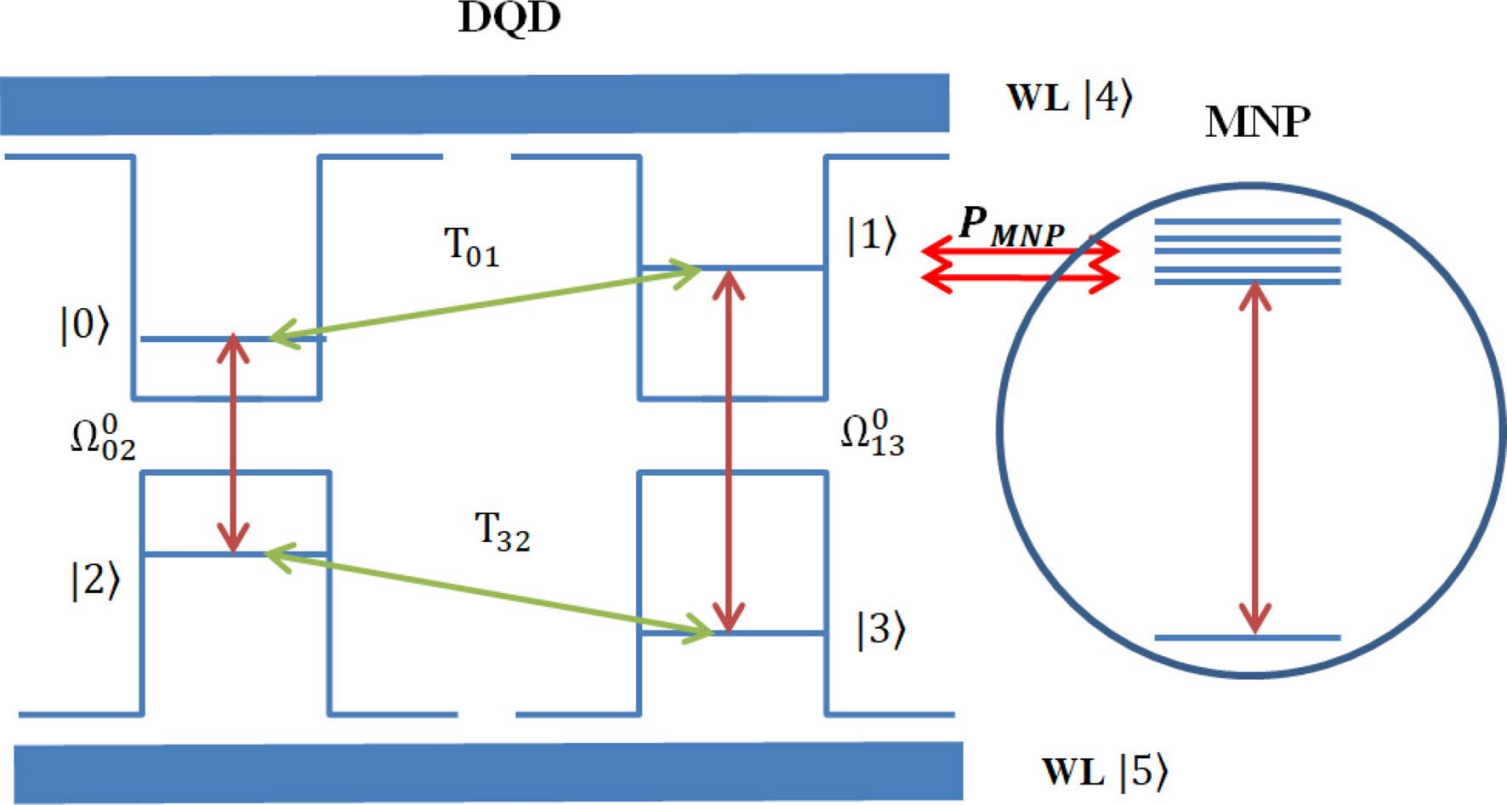
Energy band diagram for the DQD-MNP system with WL.

Consider a hybrid DQD-MNP structure with a pump and probe fields applied; see Fig. 2. A probe field ${\mathrm{E}}_{02}\left(\mathrm{t}\right)=\frac{{E}_{02}^{0}}{2}{e}^{-i{\omega }_{02}t}+\mathrm{c}.\mathrm{c}.$ with a frequency ${\omega }_{02}$ and amplitude ${E}_{02}^{0}$ is applied between $|0\rangle \leftrightarrow |2\rangle$ DQD states. Similarly, a pump laser field ${\mathrm{E}}_{13}\left(\mathrm{t}\right)=\frac{{E}_{13}^{0}}{2}{e}^{-i{\omega }_{13}t}+\mathrm{c}.\mathrm{c}.$ with a frequency ${\omega }_{13}$ and amplitude ${E}_{13}^{0}$ is applied between $|1\rangle \leftrightarrow |3\rangle$ DQD states. The Hamiltonian of the system can be written as,

1 $$H={\mathrm{H}}_{0}+{\mathrm{H}}_{int}+{\mathrm{H}}_{relax}$$

where is ${\mathrm{H}}_{0}$ the unperturbed Hamiltonian, ${\mathrm{H}}_{0}=\sum_{i=0}^{5}\mathrm{\hslash }{\upomega }_{\mathrm{i}}$ and the relaxation Hamiltonian is ${\mathrm{H}}_{relax}$. In this work, the MNP-DQD interaction Hamiltonian ${H}_{int}$ is

2 $${H}_{int}=\left[\begin{array}{ccc}0& {T}_{10}& {\Omega }_{20}\\ {T}_{10}& 0& {\beta }_{21}\\ \begin{array}{c}{\Omega }_{20}\\ {\beta }_{30}\\ \begin{array}{c}{\beta }_{40}\\ 0\end{array}\end{array}& \begin{array}{c}{\beta }_{21}\\ {\Omega }_{31}\\ \begin{array}{c}{\beta }_{41}\\ 0\end{array}\end{array}& \begin{array}{c}0\\ {T}_{23}\\ \begin{array}{c}0\\ {\beta }_{52}\end{array}\end{array}\end{array} \begin{array}{ccc}{\beta }_{30}& {\beta }_{40}& 0\\ {\Omega }_{31}& {\beta }_{41}& 0\\ \begin{array}{c}{T}_{23}\\ 0\\ \begin{array}{c}0\\ {\beta }_{53}\end{array}\end{array}& \begin{array}{c}0\\ 0\\ \begin{array}{c}0\\ {\beta }_{54}\end{array}\end{array}& \begin{array}{c}{\beta }_{52}\\ {\beta }_{53}\\ \begin{array}{c}{\beta }_{54}\\ 0\end{array}\end{array}\end{array}\right]+\left[\begin{array}{ccc}0& 0& {\mathrm{G}}_{20}\\ 0& 0& 0\\ \begin{array}{c}{\mathrm{G}}_{20}\\ 0\\ \begin{array}{c}0\\ 0\end{array}\end{array}& \begin{array}{c}0\\ {\mathrm{G}}_{31}\\ \begin{array}{c}0\\ 0\end{array}\end{array}& \begin{array}{c}0\\ 0\\ \begin{array}{c}0\\ 0\end{array}\end{array}\end{array} \begin{array}{ccc}0& 0& 0\\ {\mathrm{G}}_{31}& 0& 0\\ \begin{array}{c}0\\ 0\\ \begin{array}{c}0\\ 0\end{array}\end{array}& \begin{array}{c}0\\ 0\\ \begin{array}{c}0\\ 0\end{array}\end{array}& \begin{array}{c}0\\ 0\\ \begin{array}{c}0\\ 0\end{array}\end{array}\end{array}\right]\left[{\rho }_{ij}\right]$$

where ${\mathrm{T}}_{01}$ and ${\mathrm{T}}_{32}$ represents the tunneling components, ${\beta }_{ij}=\frac{{\mathrm{A}}_{\mathrm{ij}}}{2}+\frac{1}{{\uptau }_{t}}$, with ${\mathrm{A}}_{\mathrm{ij}}(=\frac{{{\mu }_{ij}}^{2}{\omega }_{ij}^{2}}{3\pi \hslash {\varepsilon }_{s}{c}^{3}})$ is the Einstein coefficient, ${\uptau }_{t}$ is the dipole dephasing time, ${\omega }_{ij}$ is the transition frequency between QD $|\mathrm{i}\rangle$ and $|\mathrm{j}\rangle$ states, ${G}_{ij}$ is the self-interaction of the DQD, ${\mu }_{ij}$ is the QD transition momentum between $|\mathrm{i}\rangle$ and $|\mathrm{j}\rangle$ states and ${\rho }_{ij}$ is the DQD density matrix operator.

The total electric field ${(\mathrm{E}}_{DQD,ij})$ felt by the DQD results from the superposition of the external field with induced polarization of the MNP field. It is given by,

3 $${E}_{DQD,ij}=\frac{1}{{\varepsilon }_{effs}}\left({E}_{ij}+\frac{1}{4\pi {\varepsilon }_{B}}\frac{{\delta }_{\alpha }{P}_{MNP,ij}}{{R}^{3}}\right)$$

The induced polarization of the MNP is defined as^29^,

4 $${P}_{MNP,ij}=\left(4\pi {\varepsilon }_{B}\right){a}^{3}{\upgamma }_{M} {E}_{MNP,ij}$$

With ${\upgamma }_{M}=\frac{{\upvarepsilon }_{\mathrm{M}}\left(\upomega \right)-{\upvarepsilon }_{\mathrm{B}}}{2{\upvarepsilon }_{\mathrm{B}}+{\upvarepsilon }_{\mathrm{M}}\left(\upomega \right)}$. The electric field felt by the MNP ${(E}_{MNP,ij})$ is the sum of the applied field plus the field due to the polarization of the DQD, i.e.,^3^,

5 $${E}_{MNP,ij}=\left({E}_{ij}+\frac{1}{4\pi {\varepsilon }_{B}}\frac{{\delta }_{\alpha }{P}_{DQD,ij}}{{R}^{3}}\right)$$

whereas the DQD polarization is as follows

6 $${P}_{DQD,ij}={\mu }_{\mathrm{ij}}\left({\rho }_{ij}{e}^{-i{\omega }_{ij}t}+{\rho }_{ij}^{*}{e}^{i{\omega }_{ij}t}\right)$$

From Eqs. (3)–(6), ${E}_{DQD,ij}$ becomes,

7 $${E}_{DQD,ij}=\frac{\mathrm{\hslash }}{{\mu }_{ij}}\left[\left({\Omega }_{ij}+{G}_{ij}{\rho }_{ij}\right){e}^{-i{\omega }_{ij}t}+\left({\Omega }_{ij}^{*}+{G}_{ij}^{*}{\rho }_{ij}^{*}\right){e}^{i{\omega }_{ij}t}\right]$$

where $ij$ represents either the effective Rabi frequency of the probe ${\Omega }_{02}$ or pump ${\Omega }_{13}$ field, respectively, and ${\Omega }_{ij}$ is taken by the relation^30^,

8 $${\Omega }_{ij}={\Omega }_{ij}^{0}\left(1+\frac{{a}^{3}{\upgamma }_{M} {\delta }_{\alpha }}{{R}^{3}}\right)$$

The first term of the Rabi frequency ${\Omega }_{ij}^{0}(=\frac{{E}_{ij}^{0}{\mu }_{ij}}{2\hslash {\varepsilon }_{effs}})$ is related to the direct coupling of the applied field to the DQD, while the second term is the field produced by the MNP owing to its interaction with the applied field. The parameter ${G}_{ij}$ represents the self-interaction of the DQD and is expressed as^31^,

9 $${G}_{ij}=\frac{{\upgamma }_{M} {a}^{3}}{4\pi {\varepsilon }_{B}\mathrm{\hslash }{R}^{6}}{\left(\frac{{\mu }_{ij}{\delta }_{\alpha }}{{\upvarepsilon }_{\mathrm{effs}}}\right)}^{2}$$

where ${G}_{ij}$ is produced when the applied field polarizes the DQD, which then polarizes the MNP and creates a field that interacts with the DQD^3^. From Eqs. (5) and (6) we have,

10 $${\mathrm{E}}_{\mathrm{MNP},\mathrm{ij}}= \left(\frac{{E}_{ij}^{0}}{2}+\frac{1}{4\uppi {\upvarepsilon }_{\mathrm{B}}}\frac{{\updelta }_{\mathrm{\alpha }}{\upmu }_{\mathrm{ij}}}{{\upvarepsilon }_{\mathrm{effs}}{\mathrm{R}}^{3}}{\uprho }_{\mathrm{ij}}\right){\mathrm{e}}^{-\mathrm{i}{\upomega }_{\mathrm{ij}}\mathrm{t}}+\left(\frac{{E}_{ij}^{0}}{2}+\frac{1}{4\uppi {\upvarepsilon }_{\mathrm{B}}}\frac{{\updelta }_{\mathrm{\alpha }}{\upmu }_{\mathrm{ij}}}{{\upvarepsilon }_{\mathrm{effs}}{\mathrm{R}}^{3}}{\rho }_{ij}^{*}\right){\mathrm{e}}^{\mathrm{i}{\upomega }_{\mathrm{ij}}\mathrm{t}}$$

with

11 $${E}_{MNP,\mathrm{ij}}={\widetilde{E}}_{MNP,\mathrm{ij}}{\mathrm{e}}^{-\mathrm{i}{\upomega }_{\mathrm{ij}}\mathrm{t}}+{\widetilde{E}}_{MNP,\mathrm{ij}}^{*}{\mathrm{e}}^{\mathrm{i}{\upomega }_{\mathrm{ij}}\mathrm{t}}$$

### Energy absorption rate

The absorption rate from the DQD-MNP system is introduced as^1^

12 $${Q}_{\mathrm{tot}}={Q}_{\mathrm{DQD}}+{Q}_{\mathrm{MNP}}$$

Depending on the applied fields^7^, the absorption rate in the DQD is provided by,

13 $${Q}_{\mathrm{DQD}}=\hslash {\omega }_{02}{\rho }_{00}{\gamma }_{00}+\hslash {\omega }_{13}{\rho }_{11} {\gamma }_{11}$$

To calculate the energy absorbed by the MNP, take the time average of the volume integral $\int J.{E}_{MNP,tot}dV$ where $\mathrm{J}$ is the current density and ${E}_{MNP,tot}$ is the total electric field inside the MNP^2^,

14 $${E}_{MNP,tot}= \sum_{ij=\mathrm{02,13}}\frac{{\mathrm{E}}_{\mathrm{MNP},\mathrm{ij}}}{{\upvarepsilon }_{\mathrm{effM}}}=\sum_{ij=\mathrm{02,13}}\frac{{\widetilde{E}}_{MNP,\mathrm{ij}}}{{\upvarepsilon }_{\mathrm{effM}}}{\mathrm{e}}^{-\mathrm{i}{\upomega }_{\mathrm{ij}}\mathrm{t}}+\frac{{\widetilde{E}}_{MNP,\mathrm{ij}}^{*}}{{\upvarepsilon }_{\mathrm{effM}}} {\mathrm{e}}^{\mathrm{i}{\upomega }_{\mathrm{ij}}\mathrm{t}}$$

where $${\upvarepsilon }_{\mathrm{effM}}=\frac{2{\varepsilon }_{B}+{\varepsilon }_{M}}{3{\varepsilon }_{B}}$$. Then,

15 $${\mathrm{P}}_{MNP,tot}=4\uppi {\upvarepsilon }_{\mathrm{B}}\upgamma {\mathrm{a}}^{3} \left[\sum_{ij=\mathrm{02,13}}\frac{{\widetilde{E}}_{MNP,\mathrm{ij}}}{{\upvarepsilon }_{\mathrm{effM}}}{\mathrm{e}}^{-\mathrm{i}{\upomega }_{\mathrm{ij}}\mathrm{t}}+\frac{{\widetilde{E}}_{MNP,\mathrm{ij}}^{*}}{{\upvarepsilon }_{\mathrm{effM}}} {\mathrm{e}}^{\mathrm{i}{\upomega }_{\mathrm{ij}}\mathrm{t}}\right]$$

The current density $J$ is equal to the time derivative of the polarization (dipole moment per volume) of the MNP^3^,

16 $$\mathrm{J}=\frac{\partial }{\partial t}\left(\frac{{P}_{MNP,tot}}{V}\right)$$

17 $$\mathrm{J}=\frac{-i\omega \left(4\uppi {\upvarepsilon }_{\mathrm{B}}\right){\upgamma }_{M} {\mathrm{a}}^{3}}{V}{E}_{MNP,tot}$$

where $V$ is the volume of the MNP. Thus, the energy absorption rate by the MNP is equal to^1^,

18 $${Q}_{MNP}=\int J.{E}_{MNP,tot}dV$$

This gives,

19 $${Q}_{\mathrm{MNP}}=\left(4\uppi {\upvarepsilon }_{\mathrm{B}}\right) {\mathrm{a}}^{3}\upomega {\upgamma }_{M} |{{\widetilde{E}}_{MNP,tot}|}^{2}$$

### Density matrix equations of the MNP-DQD system

The equation of motion that describes the dynamics of the DQD system is written using the density matrix theory as follows^32^,

20 $${\rho }_{ij}^{\cdot }=\frac{-i}{\hslash }\left[H,{\rho }_{ij}\right]$$

With i and j refers to the $|\mathrm{i}\rangle$ and $|\mathrm{j}\rangle$ states. As in the works discussing the hybrid QD-MNP system like^2,31,33^, using Eqs. (1) and (2), the dynamical equations of the DQD system shown in Fig. 2 are listed as,

$${\rho }_{00}^{\cdot }=-{\gamma }_{0}{\rho }_{00}\,+\,i\left[{T}_{01}\left({\rho }_{10}-{\rho }_{01}\right)+\left({\Omega }_{20}+{G}_{20}{\rho }_{20}\right)\left({\rho }_{20}-{\rho }_{02}\right)+{\beta }_{30}\left({\rho }_{30}-{\rho }_{03}\right)+{\beta }_{40}\left({\rho }_{40}-{\rho }_{04}\right)\right]$$

$${\rho }_{11}^{\cdot }=-{\gamma }_{1}{\rho }_{11}\,+\, i\left[{T}_{01}\left({\rho }_{01}-{\rho }_{10}\right)+{\beta }_{21}\left({\rho }_{21}-{\rho }_{12}\right)+\left({\Omega }_{31}+{G}_{31}{\rho }_{31}\right)\left({\rho }_{31}-{\rho }_{13}\right)+{\beta }_{41}\left({\rho }_{41}-{\rho }_{14}\right)\right]$$

$${\rho }_{22}^{\cdot }=-{\gamma }_{2}{\rho }_{22} \, + \, i\left[\left({\Omega }_{20}+{G}_{20}{\rho }_{20}\right)\left({\rho }_{02}-{\rho }_{20}\right)+{\beta }_{21}\left({\rho }_{12}-{\rho }_{21}\right)+{T}_{32}\left({\rho }_{32}-{\rho }_{23}\right)+{\beta }_{52}\left({\rho }_{25}-{\rho }_{52}\right)\right]$$

$${\rho }_{33}^{\cdot }=-{\gamma }_{3}{\rho }_{33}\, + \,i\left[{{\beta }_{30}\left({\rho }_{03}-{\rho }_{30}\right)+\left({\Omega }_{31}+{G}_{31}{\rho }_{31}\right)\left({\rho }_{13}-{\rho }_{31}\right)+T}_{32}\left({\rho }_{23}-{\rho }_{32}\right)+{\beta }_{53}\left({\rho }_{35}-{\rho }_{53}\right)\right]$$

$${\rho }_{44}^{\cdot }=-{\gamma }_{4}{\rho }_{44}\, + \,i\left[{\beta }_{40}\left({\rho }_{04}-{\rho }_{40}\right)+{\beta }_{41}\left({\rho }_{14}-{\rho }_{41}\right)\right]$$

$${\rho }_{55}^{\cdot }=-{\gamma }_{5}{\rho }_{55}+i\left[{\beta }_{52}\left({\rho }_{52}-{\rho }_{25}\right)+{\beta }_{53}\left({\rho }_{53}-{\rho }_{35}\right)+{\beta }_{54}\left({\rho }_{54}-{\rho }_{45}\right)\right]$$

$${\rho }_{10}^{\cdot }={-\left({\gamma }_{0}+{\gamma }_{1}\right)\rho }_{10} \, + \, i\left[{T}_{01}\left({\rho }_{00}-{\rho }_{11}\right)+{\beta }_{21}{\rho }_{20}+\left({\Omega }_{31}+{G}_{31}{\rho }_{31}\right){\rho }_{30}+{\beta }_{41}{\rho }_{40}-\left({\Omega }_{20}+{G}_{20}{\rho }_{20}\right){\rho }_{12} \right]$$

$${\rho }_{20}^{\cdot }=\left[-\left({\gamma }_{0}+{\gamma }_{2}\right)-i{\Delta }_{20}\right]{\rho }_{20}+i\left[\left({\Omega }_{20}+{G}_{20}{\rho }_{20}\right)\left({\rho }_{00}-{\rho }_{22}\right)+{\beta }_{21}{\rho }_{10}+{T}_{32}{\rho }_{30}+{\beta }_{30}{\rho }_{23}+{\beta }_{52}{\rho }_{50}{-T}_{01}{\rho }_{21}{-\beta }_{40}{\rho }_{24}\right]$$

$${\rho }_{30}^{\cdot }={-\left({\gamma }_{0}+{\gamma }_{3}\right)\rho }_{30}\, + \, i\left[{\beta }_{30}\left({\rho }_{00}-{\rho }_{33}\right)+\left({\Omega }_{31}+{G}_{31}{\rho }_{31}\right){\rho }_{10}+{T}_{32}{\rho }_{20}{-T}_{01}{\rho }_{31}-\left({\Omega }_{20}+{G}_{20}{\rho }_{20}\right){\rho }_{32}{-\beta }_{40}{\rho }_{34}\right]$$

$${\rho }_{40}^{\cdot }=-\left({\gamma }_{0}+{\gamma }_{4}\right){\rho }_{40}\, + \, i\left[{{{\beta }_{40}\left({\rho }_{00}-{\rho }_{44}\right){-\beta }_{41}{\rho }_{10}-\left({\Omega }_{20}+{G}_{20}{\rho }_{20}\right){\rho }_{42}-\beta }_{30}{\rho }_{43}-T}_{01}{\rho }_{41}+{\beta }_{40}{\rho }_{34}\right]$$

$${\rho }_{50}^{\cdot }={-\left({\gamma }_{0}+{\gamma }_{5}\right)\rho }_{50}\, + \, i\left[{\beta }_{52}{\rho }_{20}+{\beta }_{53}{\rho }_{30}+{\beta }_{54}{\rho }_{40}+\left({\Omega }_{20}+{G}_{20}{\rho }_{20}\right){\rho }_{52}{{-\beta }_{30}{\rho }_{53}-\beta }_{40}{\rho }_{54}\right]$$

$${\rho }_{21}^{\cdot }=-\left({\gamma }_{1}+{\gamma }_{2}\right){\rho }_{21}\, + \, i\left[{\beta }_{21}\left({\rho }_{11}-{\rho }_{22}\right)-\left({\Omega }_{20}+{G}_{20}{\rho }_{20}\right){\rho }_{01}-{T}_{10}{\rho }_{20}+{T}_{32}{\rho }_{31}-\left({\Omega }_{31}+{G}_{31}{\rho }_{31}\right){\rho }_{23}-{\beta }_{41}{\rho }_{24}+{\beta }_{25}{\rho }_{51}\right]$$

$$\begin{aligned}{\rho }_{23}^{\cdot }&={-\left({\gamma }_{2}+{\gamma }_{3}\right)\rho }_{23}\, + \, i\left[{T}_{32}\left({\rho }_{33}-{\rho }_{22}\right)+\left({\Omega }_{20}+{G}_{20}{\rho }_{20}\right){\rho }_{03}\right.\\&\quad\left.-{\beta }_{03}{\rho }_{20}+{\beta }_{21}{\rho }_{13}-\left({\Omega }_{31}+{G}_{31}{\rho }_{31}\right){\rho }_{21}+{\beta }_{25}{\rho }_{53}-{\beta }_{53}{\rho }_{25}\right]\end{aligned}$$

$${\rho }_{24}^{\cdot }=-\left({\gamma }_{2}+{\gamma }_{4}\right){\rho }_{24}\, + \, i\left[\left({\Omega }_{20}+{G}_{20}{\rho }_{20}\right){\rho }_{04}-{\beta }_{04}{\rho }_{20}+{\beta }_{21}{\rho }_{14}{-\beta }_{14}{\rho }_{21}+{T}_{32}{\rho }_{34}++{\beta }_{25}{\rho }_{54}-{\beta }_{54}{\rho }_{25}\right]$$

$$\begin{aligned}{\rho }_{25}^{\cdot }&={-\left({\gamma }_{2}+{\gamma }_{5}\right)\rho }_{25}\, + \, i\left[{\beta }_{25}\left({\rho }_{55}-{\rho }_{22}\right)+\left({\Omega }_{20}+{G}_{20}{\rho }_{20}\right){\rho }_{05}\right.\\&\quad \left.+{\beta }_{21}{\rho }_{15}+{T}_{32}{\rho }_{35}{-\beta }_{35}{\rho }_{23}+{\beta }_{24}{\rho }_{45}-{\beta }_{45}{\rho }_{24}+{\beta }_{25}{\rho }_{55}\right]\end{aligned}$$

$${\rho }_{31}^{\cdot }=-\left({\gamma }_{1}+{\gamma }_{3}\right){\rho }_{31}\, + \, i\left[\left({\Omega }_{31}+{G}_{31}{\rho }_{31}\right){\left({\rho }_{11}-{\rho }_{33}\right)+{\beta }_{30}\rho }_{01}{-{T}_{01}{\rho }_{30}+\beta }_{21}{\rho }_{32}+{T}_{32}{\rho }_{21}{-\beta }_{35}{\rho }_{51}-{\beta }_{41}{\rho }_{34}\right]$$

$${\rho }_{41}^{\cdot }=-\left({\gamma }_{1}+{\gamma }_{4}\right){\rho }_{41}\, + \, i\left[{{\beta }_{41}\left({\rho }_{11}-{\rho }_{44}\right)+{\beta }_{40}\rho }_{01}{-{T}_{01}{\rho }_{40}-\beta }_{21}{\rho }_{42}+{\beta }_{45}{\rho }_{51}-\left({\Omega }_{31}+{G}_{31}{\rho }_{31}\right){\rho }_{43}\right]$$

$${\rho }_{34}^{\cdot }=-\left({\gamma }_{3}+{\gamma }_{4}\right){\rho }_{34}\, + \, i\left[{{\beta }_{40}\rho }_{03}+{\beta }_{41}{\rho }_{13}-{\beta }_{03}{\rho }_{40}+\left({\Omega }_{31}+{\mathrm{G}}_{31}{\uprho }_{31}\right){\rho }_{41}-{{T}_{32}\rho }_{42}\right]$$

$${\rho }_{35}^{\cdot }=-\left({\upgamma }_{3}+{\upgamma }_{5}\right)\uprho _{35}+\mathrm{i}\left[{{\upbeta }_{30}\uprho }_{05}+{\mathrm{T}}_{32}{\uprho }_{25}-{\upbeta }_{25}{\uprho }_{32}+{\upbeta }_{35}{\uprho }_{55}+\left({\Omega }_{31}+{\mathrm{G}}_{31}{\uprho }_{31}\right){\uprho }_{15}+{\upbeta }_{35}\left({\uprho }_{55}-{\uprho }_{33}\right)\right]$$

21 $${\rho }_{51}^{\cdot }={-\left({\gamma }_{1}+{\gamma }_{5}\right)\rho }_{51}\, + \, i\left[{{\beta }_{52}\rho }_{21}-{T}_{01}{\rho }_{50}{-\left({\Omega }_{31}+{G}_{31}{\rho }_{31}\right){\rho }_{53}+\beta }_{53}{\rho }_{31}-{\beta }_{21}{\rho }_{52}+{\beta }_{54}{\rho }_{41}{-\beta }_{41}{\rho }_{41}\right]$$

With the condition,

$${\rho }_{00}+{\rho }_{11}+{\rho }_{22}+{\rho }_{33}=1$$

where ${\upgamma }_{\mathrm{i}}$ is the relaxation rate, ${\Delta }_{20}$ is the detuning with ${\Delta }_{20}={\upomega }_{2}-{\upomega }_{02}$, the frequency ${\upomega }_{2}$ is the resonant frequency of the 2nd DQD state, and ${\upomega }_{02}$ is the frequency difference between $|0\rangle$ and $|2\rangle$ DQD states.

### Momentum matrix elements

Calculation of the momentum matrix element ${\mu }_{ij}$ (for QD states *i* and *j*) of each interdot transition, in addition to the calculation of each WL-QD momentum matrix element ${\mu }_{iw}$ of each WL-QD transition is one of the essential features of this work. Momenta calculation is necessary because of the critical role played by the momenta in calculating the parameters of optical properties, especially Rabi frequencies appearing in Eqs. (7), (8), and (9), in addition to its implicit contribution to the calculation of ${\mathrm{G}}_{ij}$ and ${\Omega }_{ij}$ appear in the density matrix equations. Taking ${\mu }_{12}$ as an example^26^,

22 $${\mu }_{12}={C}_{mn}\left\{{\int }_{0}^{a}{J}_{m}\left({p}_{1}\rho \right){J}_{m}\left({p}_{2}\rho \right)e{\rho }^{2}d\rho \}{\int }_{0}^{{h}_{d}}[\mathrm{cos}\left({k}_{{z}_{1}}z\right)\mathrm{cos}\left({k}_{{z}_{2}}z\right)\right]dz{\int }_{0}^{2\pi }\frac{1}{2\pi }d\phi$$

where ${\mathrm{C}}_{\mathrm{mn}}$ is the normalization constant, ${J}_{m}\left({p}_{1}\rho \right)$ is the Bessel function in the QD-disk plane in the $\rho$-direction, $p$ is determined from the boundary conditions at the interface between the quantum disk and the surrounding material, $e$ is the electronic charge, $\rho$ is disk radius,${k}_{{z}_{i}}$ is the wavenumber for the QD state $|\mathrm{i}\rangle$ in the z-direction.

For the WL-QD transition, the momentum matrix element is defined here with an assignment for the states in the band. For example, ${\mu }_{35}$ is the momentum for the WL-QD transition in the VB. It is given by^34^,

23 $${\mu }_{35}=\left \langle {\varphi }_{QD}^{j=3}\bigg|e\overrightarrow{r}\bigg|{\varphi }_{WLv}\right \rangle$$

24 $${\mu }_{35}=\bigg\langle {\psi }_{QD}^{j=3}\bigg|e\widehat{\rho }\rho \bigg |{\psi }_{WLv} \bigg\rangle {A}_{{QD}_{z3}}{A}_{{w}_{z5}}\int \mathrm{cos}\left({k}_{{z}_{v}}z\right)\mathrm{cos}\left({k}_{{zw}_{v}}z\right)dz$$

where ${\varphi }_{QD}^{j=3}$, ${\varphi }_{WLv}$ are the total wavefunctions of the QD state $|3\rangle$ and WL VB, respectively, while ${\psi }_{QD}^{j=3}$ and ${\psi }_{WLv}$ are those in the $\rho$-direction, ${A}_{{QD}_{z3}}$, ${A}_{{w}_{z5}}$ are the normalization constants of the wavefunctions in the z-direction. Define,

25 $$\bigg\langle {\varphi }_{QD}^{j=3}\bigg|e\widehat{\rho }\rho \bigg|{\psi }_{WLv}\bigg\rangle =\frac{1}{{N}_{WL}}\bigg[\bigg\langle {\varphi }_{QD}^{j=3}\bigg|e\rho \bigg|{\psi }_{WLv}\bigg\rangle -\sum_{i=0}^{3}\bigg\langle {\varphi }_{QD}^{j=3}\bigg|e\rho \bigg |{\varphi }_{QD}^{\mathrm{i}}\bigg\rangle \bigg \langle {\varphi }_{QD}^{\mathrm{i}}|{\varphi }_{WLv}\bigg \rangle$$

Note that in Eq. (25), ${\mathrm{N}}_{\mathrm{WL},\mathrm{j}}$ is the normalization constant in the OPW^26^,

26 $${N}_{WL}=\sqrt{1-{\left|\sum_{i}\bigg\langle {\varphi }_{QD}^{i}\bigg|{\varphi }_{WL}\bigg\rangle \right |}^{2}}$$

The summation runs over all the DQD subbands. For the right-hand-side of Eq. (25), one has,

27 $$\left\langle {\varphi }_{QD}^{j=3}\left|e\rho\right |{\psi }_{WLv}\right\rangle =\frac{{C}_{mn}|e|}{\sqrt{A}}\int {J}_{m,j}\left(p\rho \right){e}^{ik\rho }{\rho }^{2}d\rho$$

28 $$\bigg\langle \varphi _{{QD}}^{{j = 3}} \bigg|e\rho \bigg |\varphi _{{QD}}^{{{\text{i}} = 2}} \bigg \rangle = C_{{mn,j}} C_{{mn,i}} \left| e \right|\smallint _{0}^{{h/2}} J_{{m,j}} \rho J_{{m,i}} \rho d\rho$$

29 $$\bigg \langle {\varphi }_{QD}^{\mathrm{i}} \bigg |{\varphi }_{WLv} \bigg \rangle =\frac{{C}_{mn}}{\sqrt{A}}\int {J}_{m,j}\left(p\rho \right){e}^{ik\rho }{\rho }^{2}d\rho$$

Then, considering ${\mu }_{14}$ as an example of the WL-QD transition in the CB. It is expressed as,

30 $${\mu }_{14}= \bigg \langle {\varphi }_{QD}^{j=1}\bigg| er \bigg |{\varphi }_{WL\mathrm{c}} \bigg \rangle$$

31 $${\mu }_{14}= \bigg \langle {\varphi }_{QD}^{j=1} \bigg |e\widehat{\rho }\rho \bigg |{\psi }_{WL\mathrm{c}} \bigg \rangle {A}_{{QD}_{z1}}{A}_{{w}_{z4}}\int \mathrm{cos}\left({k}_{{z}_{c}z}\right)\mathrm{cos}\left({k}_{{zw}_{c}z}\right)dz$$

32 $$\bigg \langle {\varphi }_{QD}^{j=1} \bigg |e\widehat{\rho }\rho \bigg |{\psi }_{WLv} \bigg \rangle =\frac{1}{{N}_{WL,j}} \bigg [ \bigg \langle {\varphi }_{QD}^{j=1} \bigg |e\rho \bigg |{\psi }_{WLv} \bigg \rangle -\sum_{i=0}^{1} \bigg \langle {\varphi }_{QD}^{j=1} \bigg |e\rho \bigg |{\varphi }_{QD}^{\mathrm{i}=0} \bigg \rangle \bigg \langle {\varphi }_{QD}^{\mathrm{i}=0}|{\varphi }_{WL\mathrm{c}} \bigg \rangle$$

With

33 $$\bigg \langle {\varphi }_{QD}^{j=1} \bigg |e\rho \bigg |{\varphi }_{QD}^{\mathrm{i}=0} \bigg \rangle ={C}_{mn,j}{C}_{mn,i}\left|e\right|{\int }_{0}^{h/2}{J}_{m,j}\rho {J}_{m,i}\rho d\rho$$

34 $$\bigg \langle {\varphi }_{QD}^{\mathrm{i}=0} \bigg |{\varphi }_{WL\mathrm{c}} \bigg \rangle =\frac{{C}_{mn}}{\sqrt{A}}\int {J}_{m,j}\left(p\rho \right){e}^{ik\rho }\rho d\rho$$

### Ethics approval

The work is not sent to any other site.

### Consent to participate

All the authors are consent to participate.

## Results and discussion

This section simulates the results of the hybrid DQD-MNP system. Other works use selected values for QD energy subbands and experimental transition momenta, making it easy to obtain results. Nevertheless, it takes results far from practice as the subband energy of QD with a specified size and shape is duplicated with the momentum value of a QD with another shape and size (as available in works). As it deals with material properties, this work begins with the calculation of QD energy subbands and then the momenta of transitions to get the results of each structure depending on its specified parameters from the beginning. The WL effect, a quasi-continuum state, on the QD transitions is viewed through the orthogonalized plane wave (OPW), which is inevitable in the QD transitions^34,35^. Such calculations are the figure of merit recognizing this work. This work uses our laboratory software (MAOUD-37) written under MATLAB. It is checked with experimental results in^36^ and used in many publications that deal with optical properties like^34,35,37,38^. Some of them deal with plasmonic nanostructures^39–41^. The parameters used in the calculations are listed in Table 1. Note that the momentum matrix elements are calculated via MAOUD-37 software using the relations in Momentum matrix elements section. The calculated QD energy subbands and the transition momenta are listed in Table 1 to make it easy to follow the results in this work.

**Table 1:** The parameters used in the calculations.

Parameter	Symbol	Value (unit)	Ref.
Relaxations of states	${\gamma }_{0}={\gamma }_{1}{=\gamma }_{2}={\gamma }_{3}={\gamma }_{4}={\gamma }_{5}$	1/(2.5 ns)	^42,43^
InAs QD dielectric constant	${\upvarepsilon }_{\mathrm{s}}$	$15.15{\varepsilon }_{0}$	^32^
Metal (Au) dielectric constant	${\upvarepsilon }_{\mathrm{M}}$	$6.9{\varepsilon }_{0}$	^44^

Momentum	Value (nm e)	Calculated QD momenta	
		Momentum	Value (nm e)
${\mu }_{10}$	2.5716	${\mu }_{25}$	0.0176
${\mu }_{20}$	0.0069	${\mu }_{35}$	0.0278
${\mu }_{30}$	0.0071	${\mu }_{14}$	0.0367
${\mu }_{32}$	2.3849	${\mu }_{04}$	0.0341
${\mu }_{31}$	0.0076	${\mu }_{21}$	0.0066

Calculated QD energy subbands			
QD CB subbands		QD VB subbands	
QD subband	Value (eV)	QD subband	Value (eV)
${E}_{c0}$	0.9626	${E}_{v2}$	− 0.3555
${E}_{c1}$	1.0288	${E}_{v3}$	− 0.3727

Accordingly, this software begins with the calculation of QD energy levels. Secondly, the effective Rabi frequencies ${\Omega }_{\mathrm{ij}}$ and ${\mathrm{G}}_{\mathrm{ij}}$ need the analysis of transition momenta using Eqs. (13)–(25). The density operators ${\rho }_{00}$ and ${\rho }_{11}$ are used in the ${Q}_{\mathrm{DQD}}$ calculation in Eq. (13). Also, ${Q}_{\mathrm{MNP}}$ is calculated in Eq. (19) through the field ${\mathrm{E}}_{\mathrm{MNP},\mathrm{ij}}$ using Eq. (14) via ${\rho }_{02}$ and ${\rho }_{13}$ as defined in Eq. (10). These density operators are obtained through the numerical solution of the density matrix Eq. (11), then, ${Q}_{\mathrm{tot}}$ is calculated. The dielectric constant of the background is ${\varepsilon }_{B}={\varepsilon }_{0}.$ For the DQD, the relaxation times $({\gamma }_{0}={\gamma }_{1}{=\gamma }_{2}={\gamma }_{3}={\gamma }_{4}={\gamma }_{5})$ taken the same for simplicity^42,43^. The experimental value of the Au bulk dielectric constant is considered the MNP dielectric constant ${\upvarepsilon }_{\mathrm{M}}$^44^. For ($R$ and $a$) values in the figures, we refer to the condition that appears in Section “Theory” above, i.e., $\left(R>a>{\rho }_{1},{\rho }_{2}\right)$^27,28^.

All figures here plot the total, the DQD, and the MNP absorption rates ${Q}_{\mathrm{tot}}$, ${Q}_{\mathrm{DQD}}$, and ${Q}_{\mathrm{MNP}}$, versus the probe detuning frequency ${\Delta }_{20}$. The importance of this abscissa is to show the behavior under the effect of the probe field. It also detects the MNP contribution from both its radius $(a)$ and DQD-MNP distance $(R)$ by considering the shift from resonance probe frequency (zero detuning, ${\Delta }_{20}=0$). The symmetry of the shape around the probe resonance also gives information about the system.

**Figure 3:**
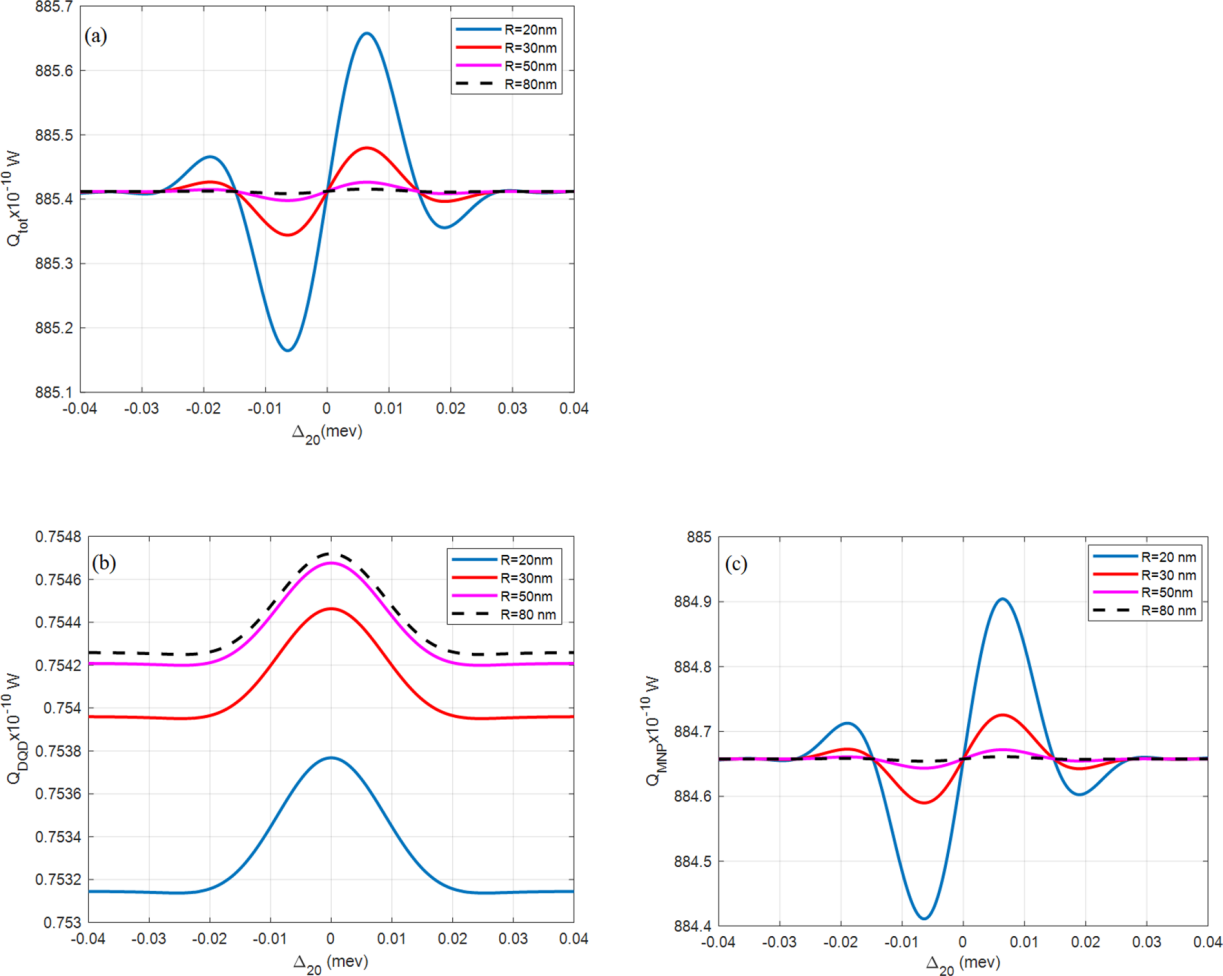
(**a**) The total $({Q}_{tot})$, (**b**) DQD (${Q}_{DQD}$), and (**c**) MNP $({Q}_{MNP})$ absorption rates from the DQD-MNP hybrid system at different spacings $(R)$ (the inset) with a = 10 nm, ${\Omega }_{02}^{0}=0.09\mathrm{ meV}$, ${\Omega }_{13}^{0}=1.8\mathrm{ GeV}$.

Figure 3 shows the total absorption rate $({Q}_{tot})$ of the DQD-MNP hybrid system at different distances (R) between DQD and MNP. The total absorption rate is increased by reducing R, which coincides with the results in^4,8^. In other works^4,8,9^, ${Q}_{tot}$ is in the range of ${10}^{-12}-{10}^{-10} W$, i.e., the absorption rate in the DQD-MNP is increased by two orders compared to QD-MNP systems. In^45^, the highest ${Q}_{tot}$ obtained is in the range ${530\times 10}^{-10} W$, i.e., the DQD-MNP structure is increased 1.6 times. This high ${Q}_{tot}$ may relate to two reasons: First, the WL washes out modes other than the condensated main mode^46^. Second, the high flexibility of manipulating DQD states compared to QD states results in more optical properties for DQD^26,34,47^. From Fig. 3, the ${Q}_{tot}$ peak is increasingly shifted at smaller DQD-MNP distance $R$, similar to the shift in the MNP rate, ${Q}_{MNP}$. This behavior results from metal proximity to the DQD structure, where the effect becomes evident at smaller distances. As the probe filed is high, the highest contribution comes from ${Q}_{MNP}$. Such behavior in QD-MNP systems is also shown in^2,33,45^. In this case, the DQD field is transferred to the MNP and then reflected in the DQD, leading to a strong enhancement of the light absorbed. However, the light enhancement is higher than the transferred energy from the QD to the MNP^47^.

**Figure 4:**
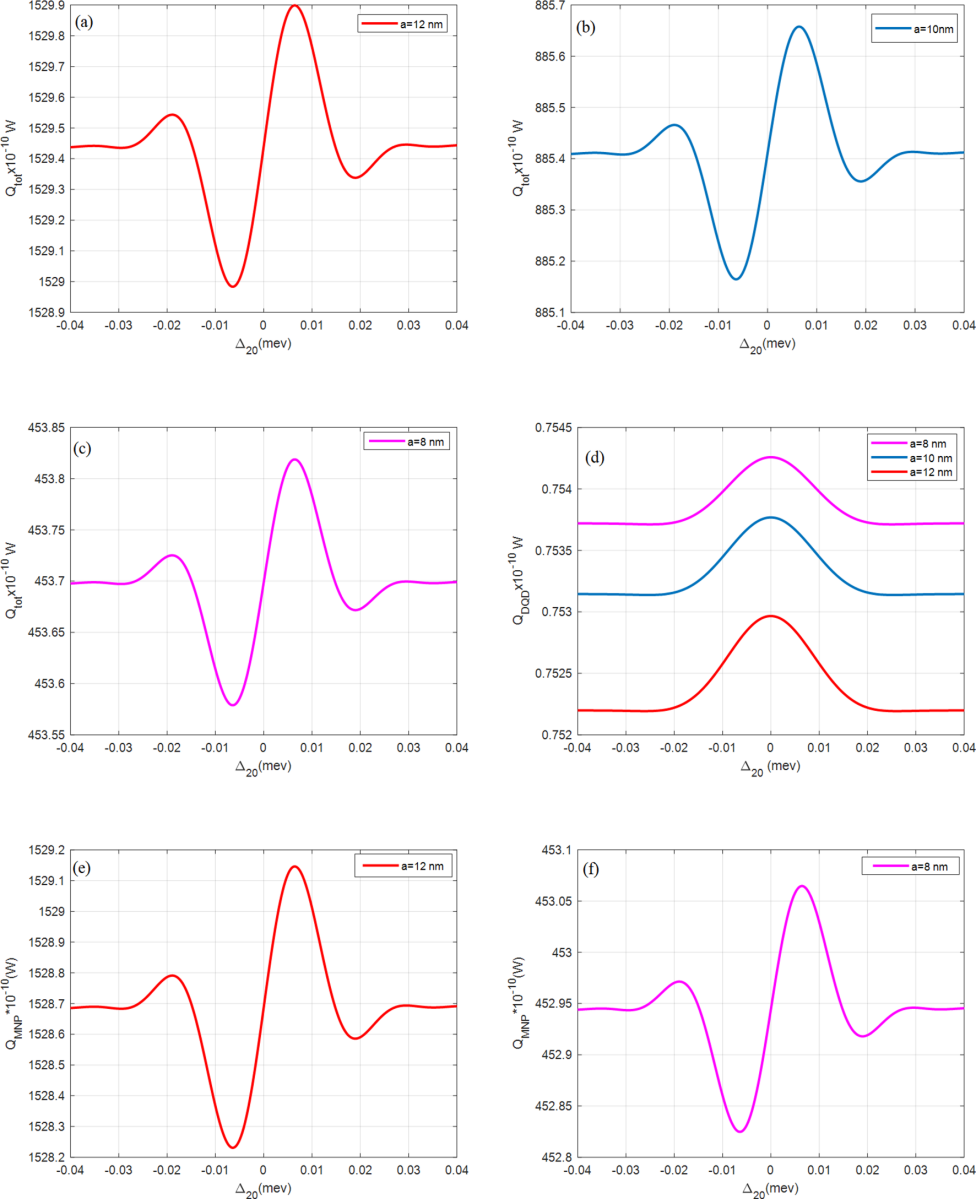
The absorption rates of the DQD-MNP hybrid system at $R=20 \, {\text{nm}}$. The total $({Q}_{tot})$ absorption rate is in (**a**) $a=12 \, {\text{nm}}$, (**b**) $a=10 \, {\text{nm}}$, and (**c**) $a=8 \, {\text{nm}}$. The DQD (${Q}_{DQD}$) is in (**d**), and then MNP $({Q}_{MNP})$ is in (**e**) $a=12 \, {\text{nm}}$, and (**f**) $a=8 \, {\text{nm}}$. In these figures ${\Omega }_{02}^{0}=0.09\mathrm{ meV}$, ${\Omega }_{13}^{0}=1.8\mathrm{ GeV},{\mathrm{T}}_{01}=30{\gamma }_{0}, {\mathrm{T}}_{32}=8{\gamma }_{0}$.

Figure 4 shows ${Q}_{tot}$ at MNP radii $a=8, 10, 12 {\text{nm}}$. The highest ${Q}_{tot}$ becomes ${1529.9\times 10}^{-10} {\text{W}}$ when $a=12 {\text{nm}}$ while for $a=10 {\text{nm}}$, the ${Q}_{tot}={885.65\times 10}^{-10} {\text{W}}$, i.e., it is increased by $0.8$ times when the MNP radius is increased by $1 \, {\text{nm}}$. The ${Q}_{DQD}$ is increased at a small MNP radius on the contrary to the ${Q}_{MNP}$ which is increased at a wider MNP radius. Since the distance $R$ is wide, a smaller MNP radius has smaller absorption, and then its ${Q}_{MNP}$. As there is more interaction at a wider MNP radius, resulting in more energy transferred to the MNP from DQD, and then the wide-radius MNP has high ${Q}_{MNP}$ while the corresponding ${Q}_{DQD}$ is reduced.

**Figure 5:**
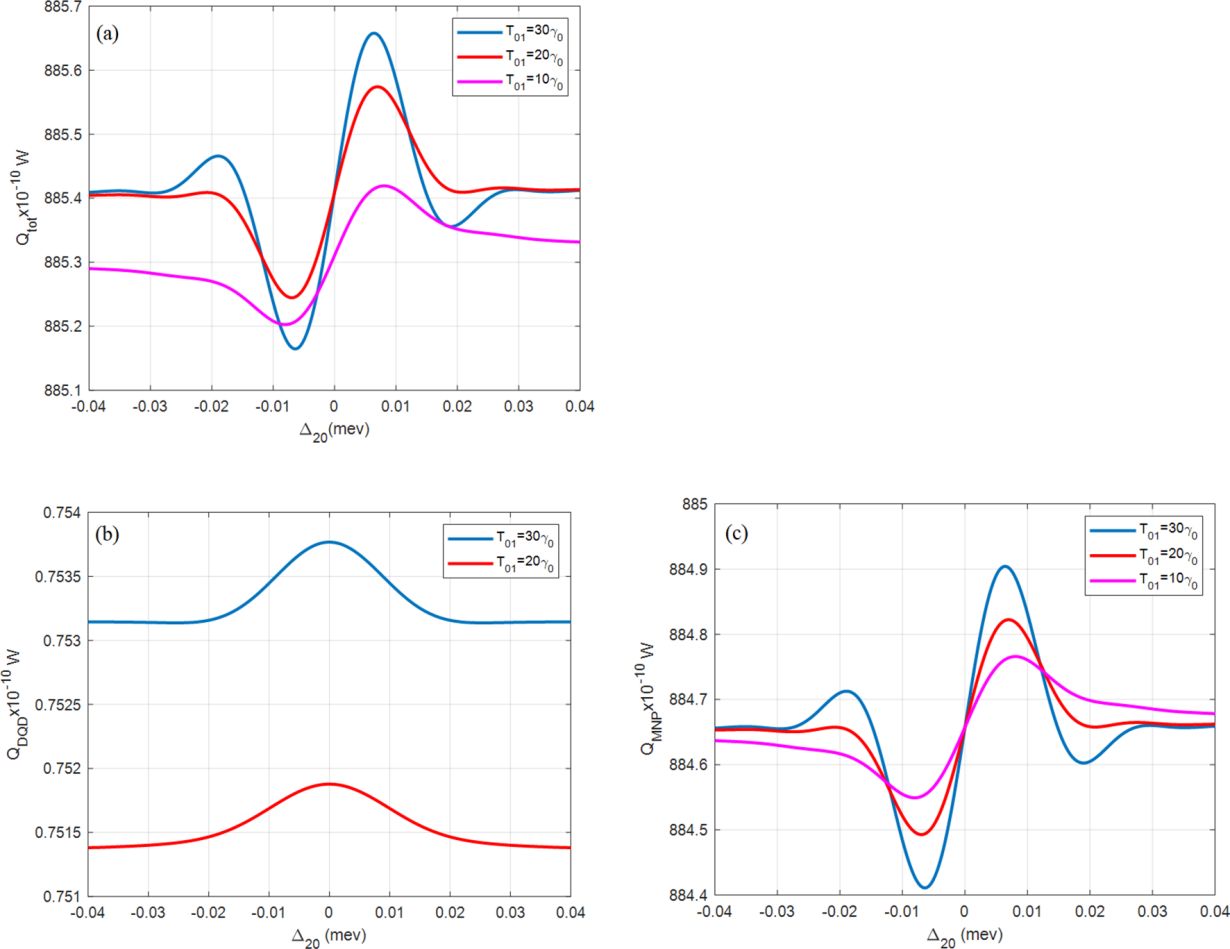
(**a**) The total $({Q}_{tot})$, (**b**) DQD (${Q}_{DQD}$), and (**c**) MNP $({Q}_{MNP})$ absorption rates from the DQD-MNP hybrid system at $R= 20 \, {\text{nm}}$ and $a=10 \, {\text{nm}}$ at different values of the tunneling component ${\mathrm{T}}_{01}$ as in the inset and ${\Omega }_{02}^{0}=0.09 \, \mathrm{ meV}$, ${\Omega }_{13}^{0}=1.8\mathrm{ GeV},{\mathrm{T}}_{32}=8{\gamma }_{0}$.

Figure 5 shows $${Q}_{tot}$$ and its components ($${Q}_{DQD}, {Q}_{MNP})$$ at different $${\mathrm{T}}_{01}$$ tunnelings. A slight red shift in the peak is exhibited with increasing tunneling. At the zero detuning, the curve is increased (in the $$Q$$ axis) by $$0.1\times {10}^{-10} \, {\text{W}}$$ at higher tunneling compared to low tunneling in the pink curve $$({T}_{10}=10{\gamma }_{0})$$. At a high electromagnetic field, there is a transition of carriers between states $$|3\rangle \leftrightarrow |1\rangle$$ and then by tunneling to the state $$|0\rangle$$ (see Fig. 1), a transfer of energy occurs to the MNP, and then a high MNP absorption occurs. This is the case in this figure and also the above ones. The curves of high tunneling $$(20{\gamma }_{0}, 30{\gamma }_{0})$$ are crossing at zero detuning while the low tunneling component (pink curve) is far from them. Checking Fig. 5b and c shows that this result comes from the difference in $${Q}_{DQD}$$.

**Figure 6:**
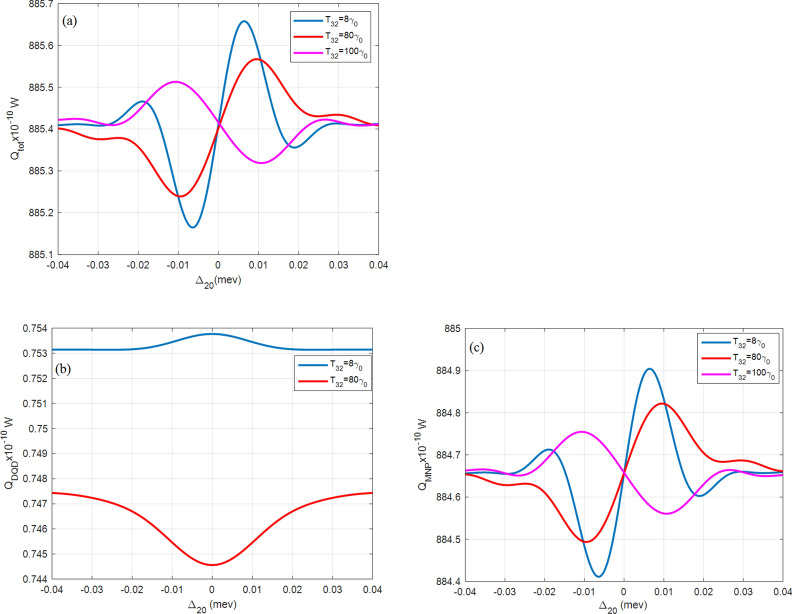
(**a**) The total $({Q}_{tot})$, (**b**) DQD (${Q}_{DQD}$), and (**c**) MNP $({Q}_{MNP})$ absorption rates from the DQD-MNP hybrid system at $R= 20 \, {\text{nm}}$ and $a=10 \, {\text{nm}}$ at different values of the tunneling component ${\mathrm{T}}_{23}$ as in the inset and ${\Omega }_{02}^{0}=0.09 \, \mathrm{ meV}$, ${\Omega }_{13}^{0}=1.8 \, \mathrm{ GeV},{\mathrm{T}}_{01}=30{\gamma }_{0}$.

Figure 6 shows ${Q}_{tot}$ at different ${\mathrm{T}}_{23}$ tunneling values. A broader blue shift than that corresponds to high ${\mathrm{T}}_{01}$ tunneling. At higher tunneling $({\mathrm{T}}_{23}=100{\gamma }_{0}$, pink curve) the curve is inverted and reduced, referring to destructive interference between fields (pump, probe, and MNP polarization field). Note that this inversion is not shown in ${Q}_{MNP}$ curves while it appears in the ${Q}_{DQD}$ at tunneling less than $100{\gamma }_{0}$ (red curve in Fig. 6b) but not affect ${Q}_{tot}$ due to the lesser contribution of ${Q}_{DQD}$ in the ${Q}_{tot}$. This result indicates that the synchronization between ${Q}_{MNP}$ and ${Q}_{DQD}$ is destroyed under high tunneling.

**Figure 7:**
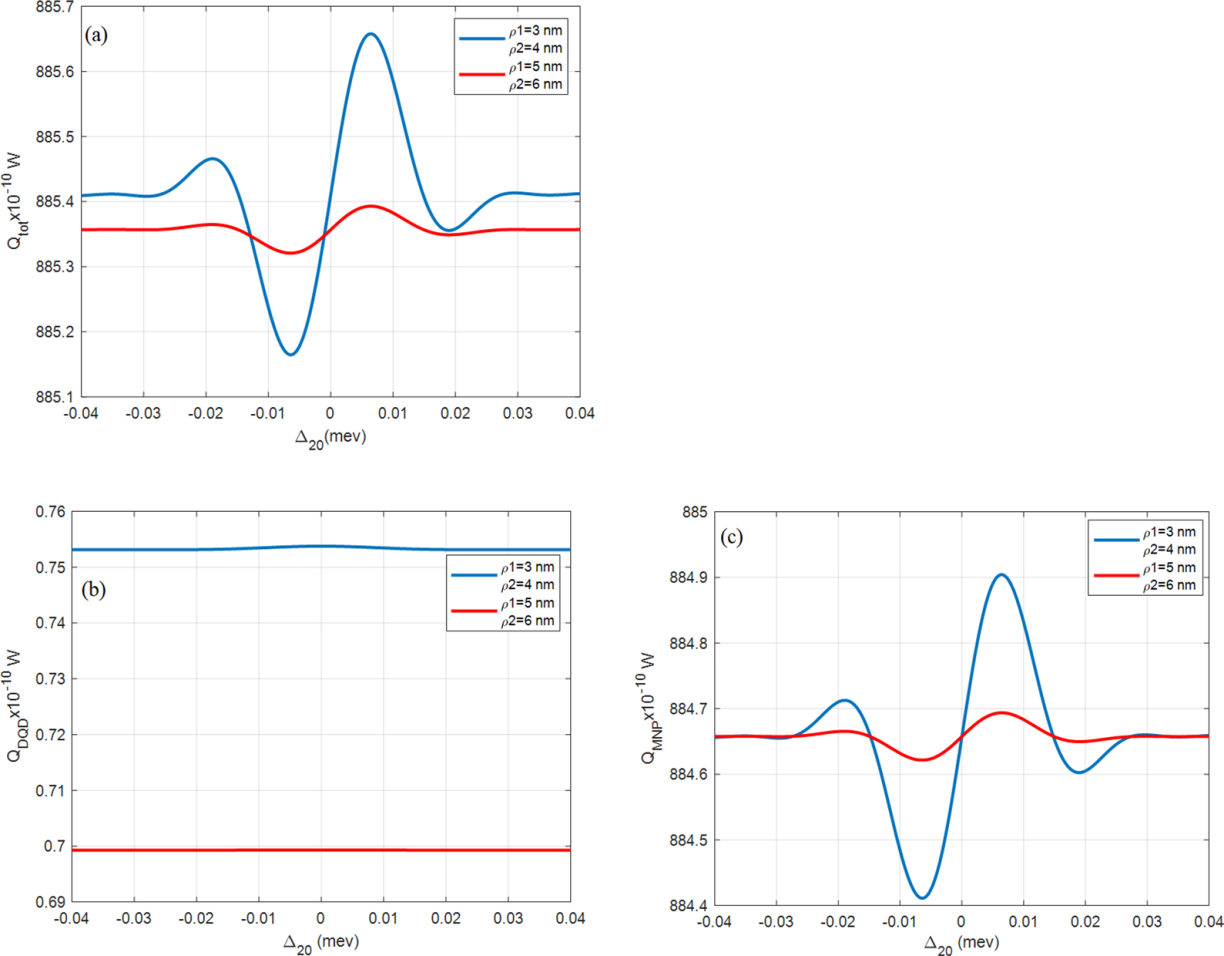
(**a**) The total $({Q}_{tot})$, (**b**) DQD (${Q}_{DQD}$), and (**c**) MNP $({Q}_{MNP})$ absorption rates from the DQD-MNP hybrid system at $R= 20 \, {\text{nm}}$ and $a=10 \, {\text{nm}}$ for different QD sizes as in the inset and ${\Omega }_{02}^{0}=0.09\, \mathrm{ meV}$, ${\Omega }_{13}^{0}=1.8\, \mathrm{ GeV},{\mathrm{T}}_{01}=30{\gamma }_{0}, {\mathrm{T}}_{32}=8{\gamma }_{0}$.

Figure 7 shows ${Q}_{tot}$ at different QD sizes. Broader QD size exhibit a high absorption rate.

**Figure 8:**
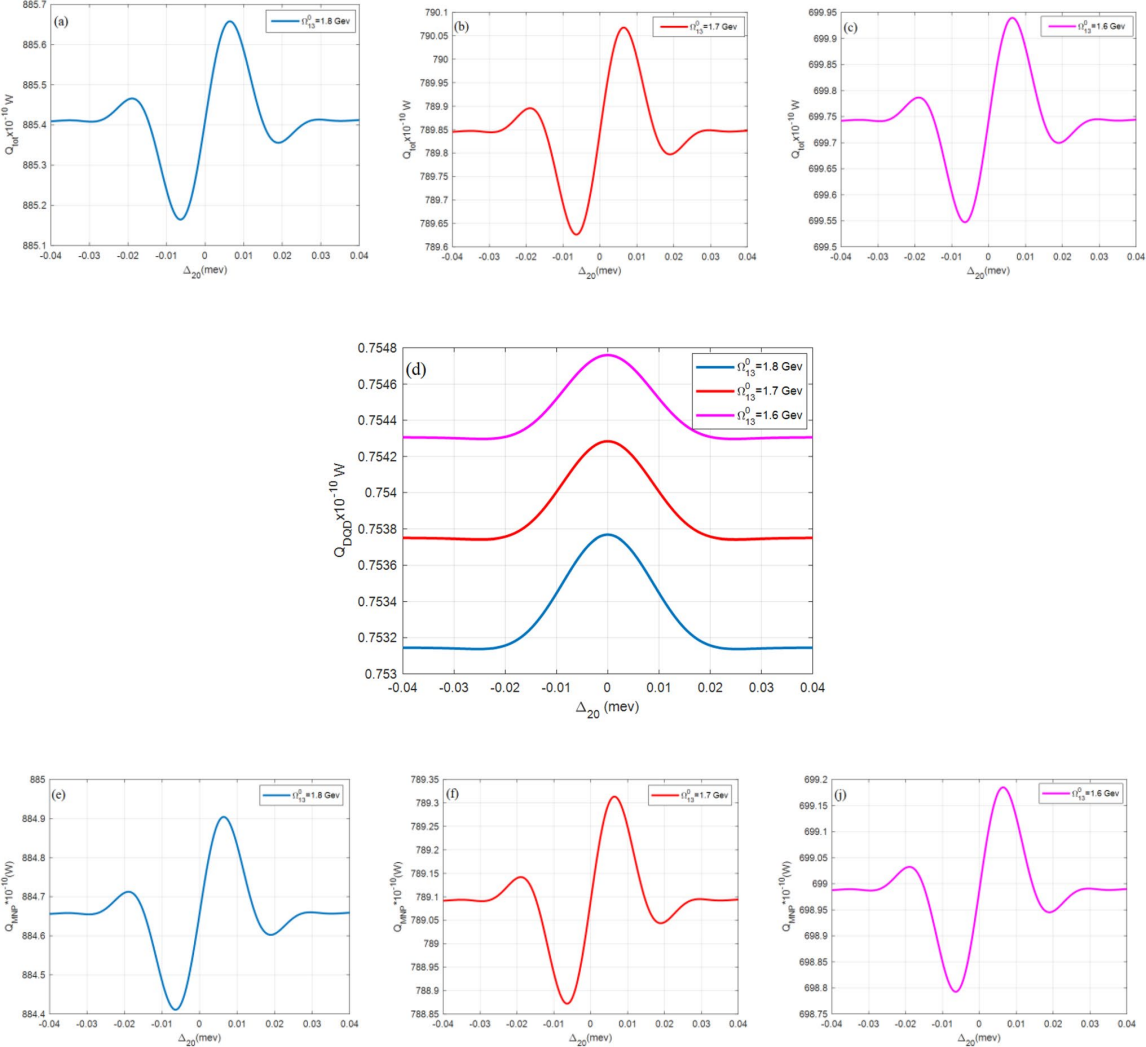
The absorption rates of the DQD-MNP hybrid system at $R= 20 \, {\text{nm}}$ under different pumpings. The total $({Q}_{tot})$ absorption rate at (**a**) ${\Omega }_{13}^{0}=1.8\, \mathrm{ GeV}$, (**b**) ${\Omega }_{13}^{0}=1.7 \, \mathrm{ GeV}$, and (**c**) ${\Omega }_{13}^{0}=1.6 \, \mathrm{ GeV}$. The DQD (${Q}_{DQD}$) is in (**d**), and then MNP $({Q}_{MNP})$ in (**e**) ${\Omega }_{13}^{0}=1.8 \, \mathrm{ GeV}$, and (**f**) ${\Omega }_{13}^{0}=1.7 \, \mathrm{ GeV}$, and (**j**) ${\Omega }_{13}^{0}=1.6 \, \mathrm{ GeV}$. All these figures are with ${\Omega }_{02}^{0}=0.09 \, \mathrm{ meV}$, ${\mathrm{T}}_{01}=30{\gamma }_{0}, {\mathrm{T}}_{32}=8{\gamma }_{0}$.

Figure 8 shows $${Q}_{tot}$$ and its components ($${Q}_{DQD}, {Q}_{MNP})$$ at different values of the pump field $$({\Omega }_{13}^{0})$$ where $${Q}_{tot}$$ and $${Q}_{MNP}$$ are increased with increasing the pump field, contrary to the $${Q}_{DQD}$$.

**Figure 9:**
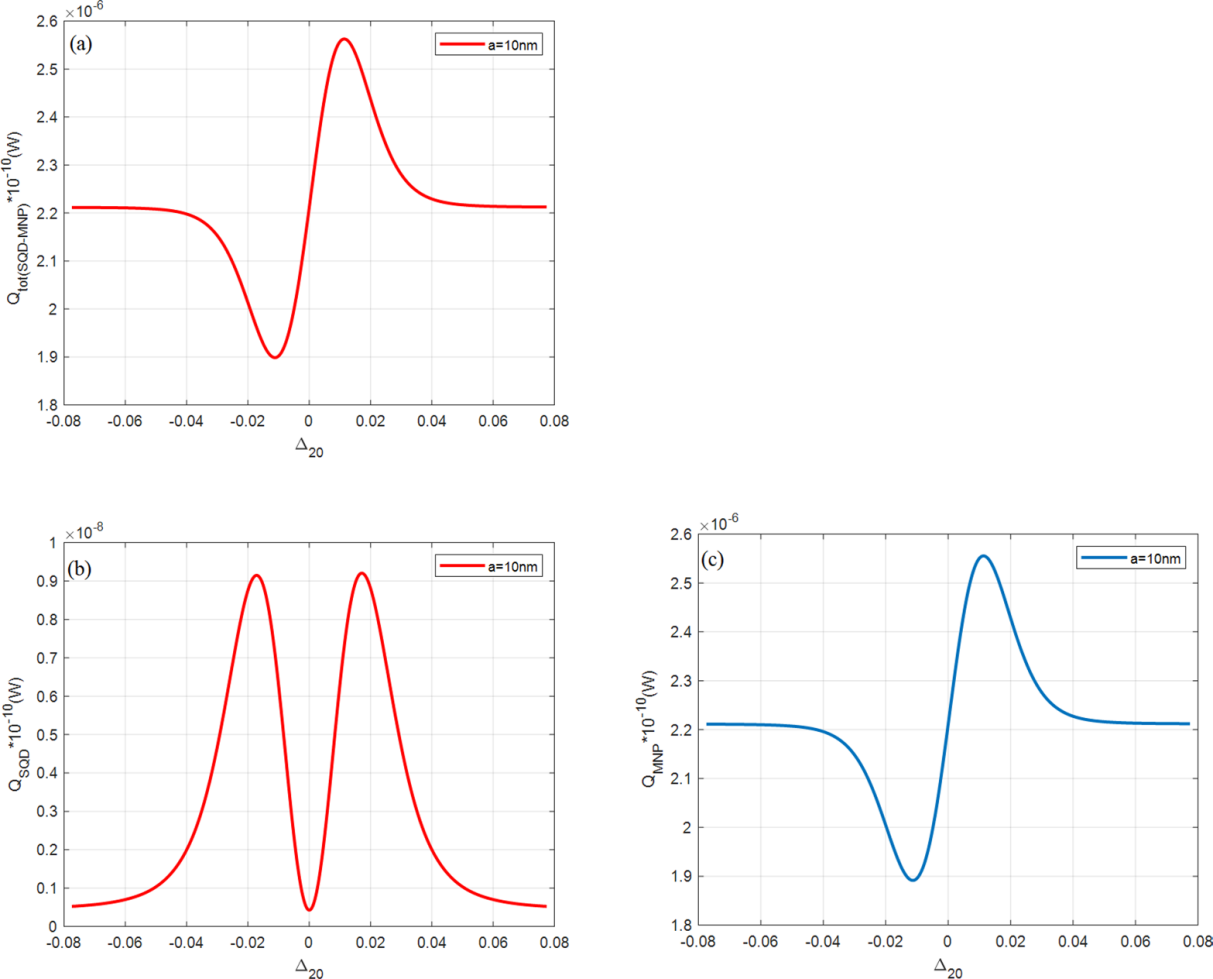
(**a**) The total $({Q}_{tot})$, (**b**) single QD (${Q}_{SQD}$), and (**c**) MNP $({Q}_{MNP})$ absorption rates from SQD-MNP hybrid system at $R= 20 \, {\text{nm}}$ and $a=10 \, {\text{nm}}$ for a single QD. Other parameters are ${\Omega }_{02}^{0}=0.09\, \mathrm{ meV}$, ${\Omega }_{13}^{0}=1.8 \, \mathrm{ GeV},{\mathrm{T}}_{01}=30{\gamma }_{0}, {\mathrm{T}}_{32}=8{\gamma }_{0}$.

Figure 9 shows $${Q}_{tot}$$ and its components for the single QD-MNP hybrid system (i.e., the absorption rate of a single QD $${(Q}_{SQD})$$ and its MNP absorption ($${Q}_{MNP})$$). Comparing this figure with the above figures (Figs. 3, 4, 5, 6, 7, 8) shows that $${Q}_{tot}$$ and $${Q}_{MNP}$$ are reduced by six orders while $${Q}_{SQD}$$ is reduced by eight orders compared to their DQD-MNP counterpart. The high absorption rate of the DQD-MNP hybrid system comes from the transition possibilities of the DQD system, which strengthens the transitions and increases the linear and nonlinear optical properties and flexibility of choosing the transitions in the DQD system^35,38,47^. This will make the DQD-MNP hybrid system preferable to QD-MNP.

**Figure 10:**
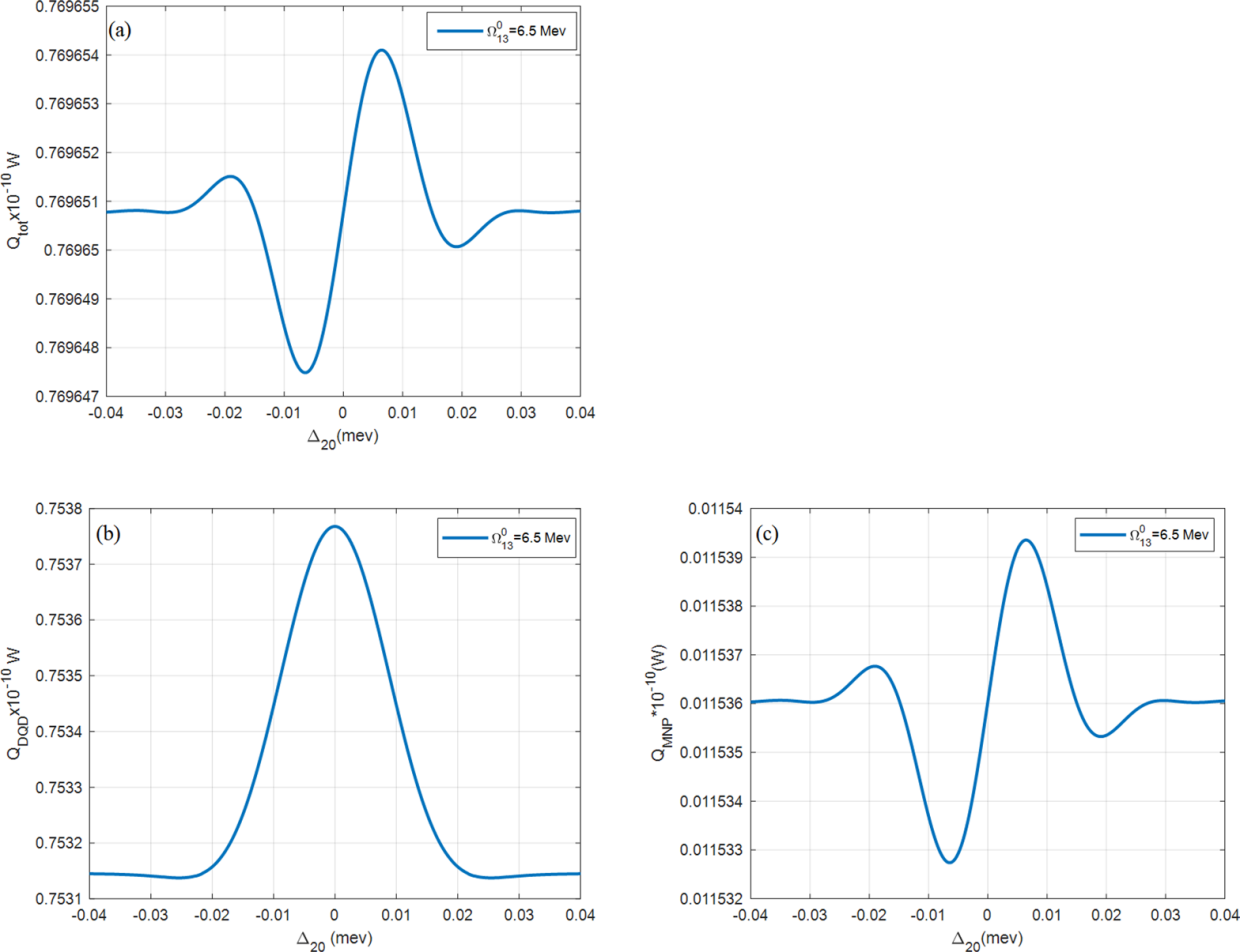
(**a**) The total $({Q}_{tot})$, (**b**) DQD (${Q}_{SQD}$), and (**c**) MNP $({Q}_{MNP})$ absorption rates from the DQD-MNP hybrid system at $R= 20 \, {\text{nm}}$ and $a=10 \, {\text{nm}}$ for DQD at a low pumping rate ${\Omega }_{13}^{0}=6.5 \, \mathrm{ MeV}$. Other parameters are ${\Omega }_{02}^{0}=0.09 \, \mathrm{ meV}$, ${\mathrm{T}}_{01}=30{\gamma }_{0}, {\mathrm{T}}_{32}=8{\gamma }_{0}$.

Finally, the absorption rates are reexamined with a low pumping field ${\Omega }_{13}^{0}$ in Fig. 10. In this case, the ${Q}_{DQD}$ is higher by more than one order than the ${Q}_{MNP}$.

## Conclusions

The double quantum dot (DQD)-metal nanoparticle (MNP) hybrid system was introduced in this work for the high energy absorption rate and modeled via the density matrix equations. The WL-DQD transitions and OPW between them are considered. The DQD energy states and momentum calculations with OPW are the figure of merit recognizing this DQD-MNP work.

The results show that at the high pump and probe fields, ${Q}_{tot}$ of the DQD-MNP hybrid system is increased by reducing R. As the probe filed is high, the highest contribution comes from ${Q}_{MNP}$. A broader blue shift at higher tunneling is seen. At low pumping field, the ${Q}_{DQD}$ is higher by more than one order than the ${Q}_{MNP}$. The broader QD size exhibits a high ${Q}_{tot}$. Compared to their single QD-MNP counterpart, ${Q}_{tot}$ and ${Q}_{MNP}$ are increased by six orders while ${Q}_{SQD}$ is reduced by eight orders. The high absorption rate of the DQD-MNP hybrid system comes from the transition possibilities of the DQD system, which strengthens the transitions and increases the linear and nonlinear optical properties and flexibility of choosing the transitions in the DQD system. This will make the DQD-MNP hybrid system preferable to QD-MNP.

## Data Availability

The data used are placed in the text of this work. All data generated or analyzed during this study are included in this work.
